# Enhanced Removal of Erythrosine B Dye Using Chemically Modified Chitosan Beads: A Comparative Evaluation

**DOI:** 10.3390/molecules31101765

**Published:** 2026-05-21

**Authors:** Fatin Aqilah Mohd Nasir, Nur Rabiatul Amierah Mohd Ariff, Zulaikha Mohd Kamal, Muhammad Adnan Iqbal, Maria Khalid, Faisal Jamil, Vikneswari Perumal, Puvana Devi Selvarajoo, Tavamani Balan, Sharon Fatinathan

**Affiliations:** 1 Faculty of Pharmacy and Health Sciences, Royal College of Medicine Perak, Universiti Kuala Lumpur, Ipoh 30450, Perak, Malaysia; fatinmnasir@gmail.com (F.A.M.N.); amierah.ariff@yahoo.com (N.R.A.M.A.); zulaikhamohdkamal@gmail.com (Z.M.K.); puvana@unikl.edu.my (P.D.S.); tavamani@unikl.edu.my (T.B.); 2 Department of Chemistry, University of Agriculture, Agriculture University Road, Faisalabad 38000, Pakistan; adnan.iqbal@uaf.edu.pk (M.A.I.); maria.khalid.uaf@gmail.com (M.K.); faisaljamilanjums@gmail.com (F.J.); 3 School of Pharmacy, Management and Science University, University Drive, Off Persiaran Olahraga, Shah Alam 40100, Selangor, Malaysia; vikneswari_perumal@msu.edu.my

**Keywords:** chitosan, crosslinking, erythrosine B (ER) dye, kinetic studies, isotherm studies, density functional theory (DFT)

## Abstract

Erythrosine B (ER) dye is widely used and increasingly detected in wastewater, necessitating effective removal. This study compares chitosan beads (CB) and chemically crosslinked beads, namely chitosan–tripolyphosphate (CT) and chitosan–sulphite (CS), for ER removal via batch adsorption studies. Characterisation confirmed successful crosslinking of the modified beads. Under optimised conditions, CB, CT, and CS achieved removal efficiencies of 75.27%, 91.69%, and 98.73%, respectively, at an initial concentration of 100 mg/L within 50–60 min. Kinetic analysis suggested that the rate-controlling step was not solely governed by intraparticle diffusion but also involved physisorption and chemisorption. While the Langmuir isotherm adequately described the adsorption process of CB and CT, with maximum adsorption capacities of 71.80 mg/g and 89.33 mg/g, respectively, a better fit was observed for the Freundlich and Redlich–Peterson isotherms, indicating multilayer adsorption. In contrast, CS showed moderate agreement with all isotherms, suggesting a complex removal process. CS demonstrated the highest adsorption capacity, with 120.30 mg/g, highlighting sodium metabisulphite (SM) as a promising crosslinking agent for improved dye removal. Density functional theory (DFT) analysis proposed that at the molecular level, interactions between the ionised oxygenated groups of ER and protonated amine groups of chitosan facilitated the adsorption process.

## 1. Introduction

Synthetic dyes are used in various industries to impart colour to distinguish products and to increase their aesthetic appeal. In recent years, there has been a growing concern about wastewater containing synthetic dyes, which possess toxicological effects [1].

**Figure 1:**
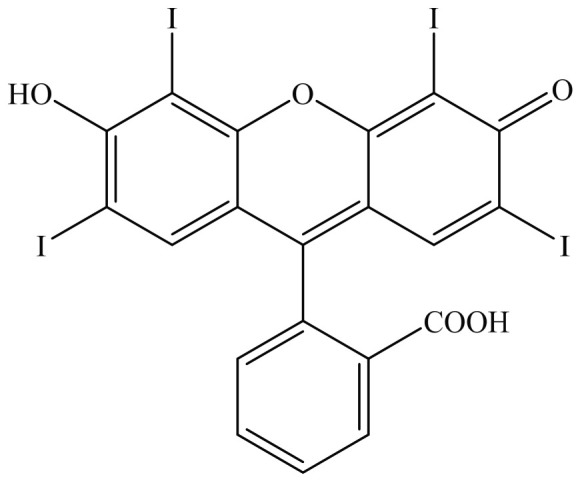
Structure of erythrosine B (ER).

One of the most widely used synthetic dyes is erythrosine B (ER), also known as Federal Food, Drug, and Cosmetic (FD&C) Red No. 3. It has four iodine atoms attached to its xanthene structure, with a molecular weight of 879.9 g/mol, and it is water-soluble. Its IUPAC name is [2-(6-hydroxy-2,4,5,7-tetraiodo-3-oxo-xanthene-9-yl) benzoic acid], and it is also symbolised with the code number E127 [2]. The chemical structure of ER is shown in Figure 1. It is used in food, cosmetics, and drug products [3]. Since 2011, the European Food Safety Authority (EFSA) has limited the acceptable daily intake (ADI) of ER dye to 0.0–0.1 mg/kg body weight, as reaffirmed in 2018 by the Joint Food and Agriculture Organization of the United Nations (FAO)/World Health Organization (WHO) Expert Committee on Food Additives (JECFA) [4]. In 2025, the Food and Drug Administration (FDA) revoked the authorisation for the use of ER in food and drug products. Industries have been given until January 2027 for food products and January 2028 for drug products to reformulate their products [5]. However, as other countries are still using this dye, there is a need to monitor the effects of ER on human and animal health. According to Singh and Chadha [6], ingestion of ER can cause damage to the gastrointestinal tract. Furthermore, exposure to ER can increase free radical activity and oxidative damage. Prolonged exposure to ER can disrupt the conversion of thyroxine (T4) to triiodothyronine (T3), leading to the development of goitre and even tumours [7,8]. Due to the complexity of its molecular structure and the intensity of its colour, ER dye poses a real challenge when it comes to its removal from water bodies [3].

Various physical and chemical treatment methods, such as ion exchange, chemical precipitation, and membrane filtration, are used to treat wastewater containing dyes. Although these techniques have advantages, they also have some drawbacks, such as reduced efficiency, high cost, limited selectivity, susceptibility to fouling, high energy demand, or sludge generation [9]. Adsorption remains a preferred method because it is low-cost, efficient, and convenient. Although many treatment centres use activated carbon (AC) as an adsorbent, it is costly, especially when the quality of the AC is higher [10]. Synthetic polymers such as polyacrylamide, polyacrylic acid, and styrene have also been widely used in wastewater treatment. However, their application has wavered over time due to their potential to leach toxic monomers, non-renewability, and greenhouse gas emissions during production [11].

As a result, researchers have explored various adsorbents made from agricultural waste, metal oxides, and aluminosilicate minerals for the removal of ER dye. Rashtbari et al. [12] reported an adsorption capacity of 144.92 mg/g for ER using zinc oxide nanoparticles loaded on AC derived from worn tyres. Meanwhile, multiwalled carbon nanotubes deposited with zinc oxide and silver oxide achieved an even higher capacity of 184.94 mg/g [13]. In contrast, biochar derived from wood chips and corn cobs showed lower adsorption capacities of 25.20 mg/g and 7.50 mg/g for ER, respectively [14]. Carbonised date stones and zeolite nanostructures with Fe-Co-V also demonstrated low adsorption capacity for ER with 9.09 mg/g and 1.82 mg/g, respectively [15,16]. Despite the promising adsorption capacities displayed by some of these adsorbents, their limitations include high cost, complicated synthesis processes, high energy consumption, and difficulty in tunability. Some of these adsorbents may also pose environmental concerns due to their limited biodegradability and the release of nanoparticles into the ecosystem [17].

Sustainable biopolymers have emerged as promising alternatives for the removal of pollutants via adsorption [11]. Researchers are extensively exploring these adsorbents due to their excellent properties, such as being non-toxic, biodegradable, and modifiable. Among naturally derived biopolymers, chitosan has been highly explored. Chitosan, primarily obtained from seafood waste, is recognised as a renewable, biodegradable, and non-toxic material [18,19]. Iqbal et al. [20] reported that, compared to synthetic polymeric resins and selected AC, chitosan-based materials offer significant sustainability advantages. Chitosan contains amine and hydroxyl functional groups that can be chemically modified [21]. These functional groups facilitate binding with pollutants through chemical interactions, while chitosan’s porous structure supports physical adsorption [22]. However, chitosan has limitations such as swelling in acidic media, low mechanical strength, and limited surface area [23]. To overcome these drawbacks, chemical crosslinking through the formation of covalent or ionic bonds can be introduced into the chitosan structure. Various crosslinking agents can be used, such as glutaraldehyde, polyethylene glycol (PEG), genipin, epichlorohydrin (ECH), and ethylene glycol diglycidyl ether (EGDE) [24]. Through crosslinking, the mechanical properties of chitosan can also be enhanced. A study by Şenol and Keskin [23] reported that a chitosan–alginate biocomposite crosslinked with ECH and sodium tripolyphosphate (STPP) exhibited an adsorption capacity of 319.00 mg/g during the removal of ER at pH 2. Chitosan modified with boric acid removed 96.50% of ER using only 0.05 g of adsorbent [25]. Chitosan can also be reversibly imprinted with ER, as studied by Eser et al. [26]. The ER-imprinted magnetic chitosan achieved a maximum adsorption capacity of 116.27 mg/g, compared to 39.06 mg/g for non-imprinted chitosan.

Most commonly used crosslinking agents tend to be harmful to humans and other life forms. Therefore, there is a need to use an environmentally friendly crosslinking agent that does not generate secondary waste that is detrimental to the environment. One such crosslinking agent is STPP [27]. STPP is recognised as safe for humans, is inexpensive, and acts as an effective crosslinking agent. The crosslinking process involves ionic interactions between the negatively charged tripolyphosphate ions and the protonated amine groups of chitosan [28]. The resulting crosslinked beads exhibit improved rigidity, pH stability, and adsorption performance. Due to its established performance, STPP was used in this study as a benchmark for comparison. Furthermore, a low degree of crosslinking was used to preserve the active functional groups involved in the adsorption process.

In addition, sodium metabisulphite (SM) was used in this study as an alternative crosslinking agent. SM is widely used in the food industry for its antioxidant and antibacterial properties [29]. However, its application in the adsorption of dyes is very limited. As sulphur is more electronegative than phosphorus, the ionic interactions between chitosan and sulphur are expected to be stronger than those formed between chitosan and phosphorus. This can enhance the binding affinity and mechanical strength of the beads. In addition, sulphite groups may introduce additional functional groups that improve the adsorption capacity. Some studies have found that sulphur-containing chitosan has a high affinity for metal ions, consistent with the hard–soft acids and bases (HSAB) theory [30]. However, research on sulphur-functionalised chitosan as a dye adsorbent remains limited. This study, therefore, explores the performance of chitosan crosslinked with SM alongside the well-established STPP.

This study compares the adsorption performance of chitosan (CB), chitosan–tripolyphosphate (CT), and chitosan–sulphite (CS) beads in the removal of ER via batch adsorption studies. The incorporation of SM as a crosslinking agent introduces an alternative modification that is yet to be extensively explored in adsorption studies. Critical parameters influencing the adsorption capacity, including the initial pH, agitation period, and adsorbent dosage, were investigated and optimised. Kinetic and isotherm models were utilised to characterise the adsorption behaviour and to elucidate the differences in performance among the three beads. The adsorption capacities were further supported using density functional theory (DFT), providing molecular-level insights that complement experimental data and validate the proposed interaction mechanisms. Through this integrated experimental and theoretical approach, this study establishes a structure–interaction relationship governing ER adsorption. These findings contribute to the development of sustainable chitosan-based adsorbents in alignment with United Nations Sustainable Development Goal 6.

## 2. Results and Discussion

### 2.1. Characterisation of CB, CT and CS Beads

**Figure 2:**
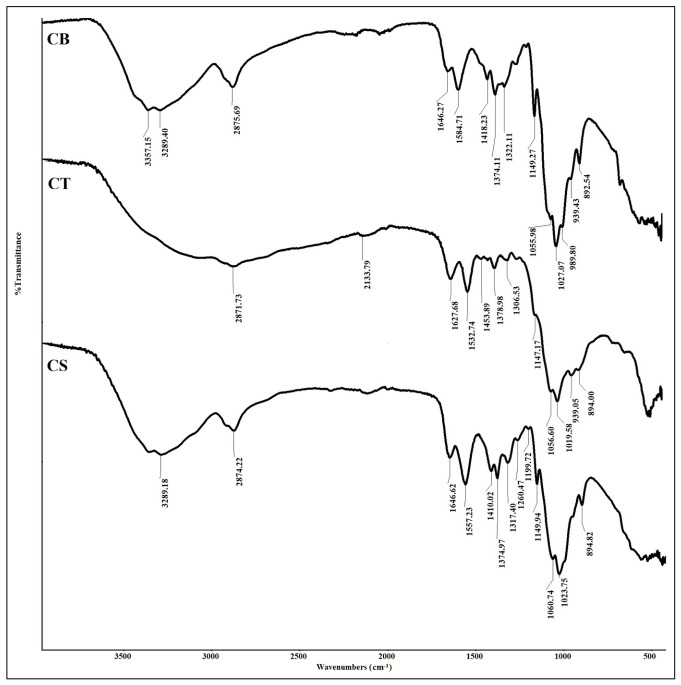
Fourier transform infrared (FTIR) spectra of chitosan (CB), chitosan–tripolyphosphate (CT), and chitosan–sulphite (CS) beads.

The Fourier transform infrared (FTIR) spectra of CB, CT, and CS beads are presented in Figure 2. In the CB beads spectrum, the broad absorption band between 3357 cm^−1^ and 3289 cm^−1^ is attributed to overlapping stretching vibrations of -OH and -NH bonds, corresponding to the hydroxyl and amine functional groups in the chitosan backbone. The peak at 2875 cm^−1^ is associated with asymmetric and symmetric aliphatic C-H stretching vibrations [31]. The band at 1646 cm^−1^ is assigned to the C=O stretching of residual amide groups, which persists due to the low degree of deacetylation of chitosan, while the band at 1584 cm^−1^ is related to the bending of the primary N-H bond [32,33]. The band at 1418 cm^−1^ corresponds to N-H deformation vibrations in -NH_2_, whereas the peak at 1374 cm^−1^ is attributed to C-N stretching [34]. The asymmetrical stretching vibrations of C-O-C from the glycosidic linkages are observed at 1149 cm^−1^. In addition, the symmetrical stretching of glycosidic linkages appears at 1027 cm^−1^ and 989 cm^−1^, while the band at 892 cm^−1^ is characteristic of the saccharide structure of chitosan [31].

Following crosslinking, the FTIR bands of CT and CS beads exhibited noticeable shifts towards lower wavenumbers, suggesting interactions between chitosan functional groups and the crosslinking agents [35]. A shift in the amide band from 1646 cm^−1^ to 1627 cm^−1^, accompanied by increased intensity, supports the occurrence of crosslinking involving the C=O groups. The band at 1418 cm^−1^ (N-H deformation vibrations in -NH_2_) diminished in the CT spectrum, while a new band emerged at 1378 cm^−1^, consistent with interactions between phosphate groups and protonated chitosan [36]. A prominent band at 1532 cm^−1^ is attributed to protonated amine groups (-NH_3_^+^), which facilitate ionic interactions with the negatively charged tripolyphosphate groups [37,38]. The weak peak at 1147 cm^−1^ is associated with P=O stretching, whereas the band at 894 cm^−1^ corresponds to P-O vibrations [39]. Additionally, a subtle shoulder near 1097 cm^−1^ suggests the presence of P=O groups [40].

The CS beads spectrum displayed variations in both peak intensity and position, confirming interactions between chitosan and SM [41]. Similar to the CT beads, a strong band at 1557 cm^−1^ is assigned to protonated amine groups, indicating that -NH_2_ groups were protonated during the crosslinking process. Moreover, the C=O band of the amide groups showed reduced intensity compared to CT beads. A distinct peak at 1317 cm^−1^ corresponds to the sulphonamide group (S=O), confirming the successful crosslinking of chitosan with SM [42].

The CB beads exhibited a Brunauer–Emmett–Teller (BET) surface area of 0.80 m^2^/g, while the Langmuir surface area was slightly higher at 1.02 m^2^/g. The average pore diameter determined by the BET method was 71.82 Å. In contrast, the CT beads showed reduced surface characteristics, with BET and Langmuir surface areas of 0.48 m^2^/g and 0.58 m^2^/g, respectively, along with a smaller average pore diameter of 47.81 Å. This observation is consistent with the findings reported by Babakhani and Sartaj [27], where chitosan crosslinked with STPP exhibited a reduction in both surface area and pore size. Meanwhile, the CS beads demonstrated the highest surface area among the three beads, with BET and Langmuir values of 0.84 m^2^/g and 1.09 m^2^/g. Coincidentally, the CS beads possessed the smallest BET average pore diameter at 41.21 Å. The higher surface area and smaller pore size suggest an enhanced adsorption of ER. These results indicate that the use of SM as the crosslinking agent improves the textural properties of the beads compared to STPP. Based on the International Union of Pure and Applied Chemistry (IUPAC) classification, all samples fall within the mesoporous range [43]. Variations in the surface area and pore size clearly indicate that different crosslinking strategies significantly influenced the structural properties of the beads. 

**Figure 3:**
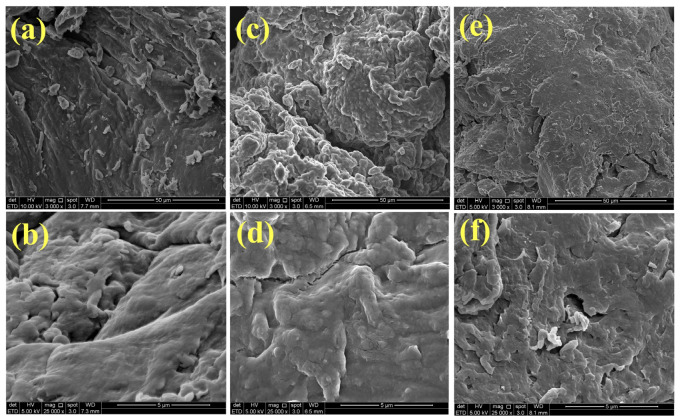
Field emission scanning electron microscope (FESEM) of (**a**,**b**) CB, (**c**,**d**) CT, and (**e**,**f**) CS beads at 3000× magnification and 25,000× magnification.

The surface morphology analysis shown in Figure 3 revealed that CB and CT beads possess rough and flaky surfaces. In comparison, the CS beads displayed a relatively smoother yet denser surface with fewer irregularities. At higher magnification, all beads exhibited microfractures and the presence of pores. These pores and microcracks facilitate enhanced adsorption [44].

**Figure 4:**
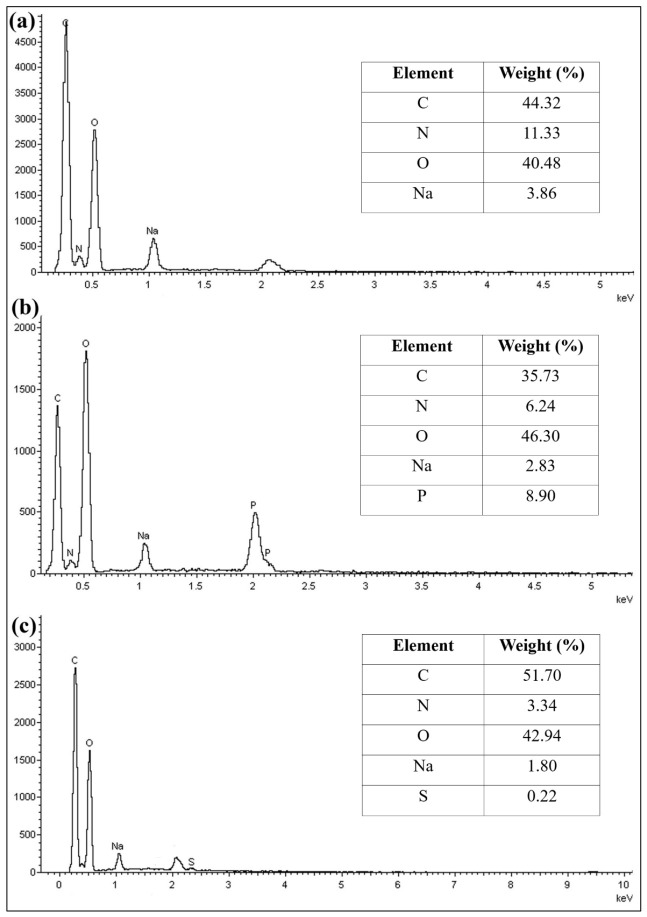
Energy dispersive spectroscopy (EDS) spectra of (**a**) CB, (**b**) CT, and (**c**) CS beads.

Energy dispersive spectroscopy (EDS) analysis (Figure 4) indicated a noticeable decrease in carbon content and an increase in oxygen content following crosslinking with STPP. The elevated oxygen content confirms successful crosslinking, as STPP is rich with oxygen-containing groups. In contrast, the CS beads exhibited an increase in carbon content, with a marginal rise in oxygen content. The slight increase in oxygen is consistent with the contribution from SM. Both crosslinked beads showed a reduction in nitrogen content compared to CB beads. The presence of phosphorus and sulphur elements in CT and CS beads further confirms the successful incorporation of the respective crosslinking agents. Sodium was detected in CB due to the use of sodium hydroxide (NaOH) during bead formation. As the crosslinking agents used are sodium-based salts, sodium remained detectable in the crosslinked beads.

### 2.2. Batch Adsorption Studies on the Adsorption of ER

#### 2.2.1. Effect of Initial pH of ER Solution

Based on the ultraviolet (UV)–visible full scan spectrum, ER showed the highest λ_max_ at 527 nm, correlating with the existence of a xanthene core in its structure. The presence of the four iodine atoms in its structure gives the dye its characteristic colour. ER dye has two pKa values at 3.9 and 5.0, corresponding to the ionisation of the phenolic (O^−^) and carboxylic (COO^−^) groups, respectively [45]. The absorbance readings of ER decreased significantly at pH values below 4.0 and above 10.0. According to Groeneveld et al. [46], the pH of the dye solution influences the chemical structure and the stability of the dye. At certain pH values, the dye molecules will aggregate, thus reducing the intensity of the dye. At pH < 5.0, the dye solution appeared brownish, with precipitation observed at pH 1, corresponding to the reduced absorbance reading. Similarly, at pH > 10, red precipitates were formed. Such precipitation lowers the measured absorbance readings and may lead to misinterpretation during the adsorption studies.

**Figure 5:**
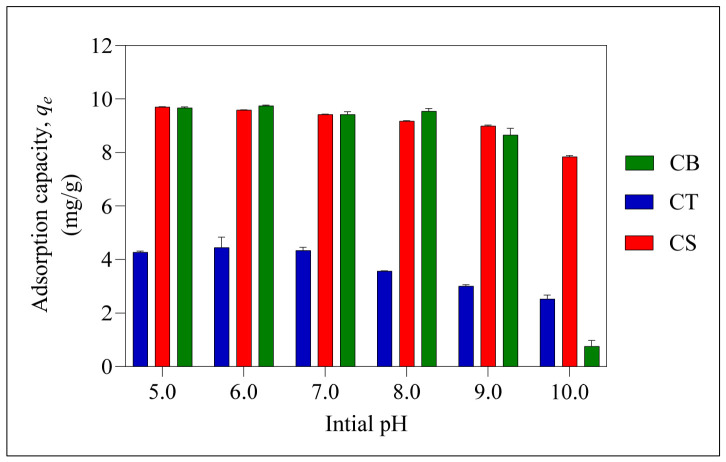
Influence of initial pH on the adsorption of ER. Error bars represent the standard deviation obtained for triplicate measurements.

Figure 5 shows that as the initial pH moves from acidic to alkaline conditions, the adsorption capacity for all beads increases and then gradually decreases. At pH > 5, ER exists predominantly in its ionised form (O^−^ and COO^−^), while the amine groups on chitosan are protonated to -NH_3_^+^, facilitating either electrostatic interactions or hydrogen bonding [47]. At a higher pH, the amine groups found in CB beads are not protonated, therefore reducing the interactions, leading to decreased adsorption. In addition, the reduced adsorption capacity, *q_e_*, of CB beads under alkaline conditions is attributed to the swelling effect caused by the disruption of intermolecular hydrogen bonding, which increases water uptake and subsequently reduces the adsorption efficiency [48].

For CT beads, the uncrosslinked -NH_2_ groups can still undergo protonation and interact via similar electrostatic interaction or hydrogen bonding with the deprotonated ER molecules. However, the adsorption capacity attained was lower than that of CB, as a significant number of the amine groups were involved in the crosslinking process with tripolyphosphate ions (P_3_O_10_^5−^). Although adsorption decreased with increasing pH, CT exhibited a higher removal efficiency than CB at pH 10.0. This is attributed to the reduced flexibility of the polymer chain after crosslinking, which promotes adsorption through diffusion within the mesoporous network [48,49].

CS beads demonstrated exceptional removal efficiency across the entire pH range studied. This implies that SM effectively reinforced the polymer network, mitigating the swelling effect at an alkaline pH. Furthermore, the relatively low crosslinking percentage preserves a high number of -NH_2_ groups for protonation, thereby enhancing the electrostatic interactions with the negatively charged ER or even the hydrogen bonding. The presence of mesopores in all three beads also enhances adsorption. The CB and CS beads, which exhibited higher surface areas, showed higher adsorption capacity compared to the CT beads, which had a lower surface area [50]. Overall, the optimum initial pH for the adsorption of ER by all three beads was found to be at pH 6.

#### 2.2.2. Effect of Agitation Period

**Figure 6:**
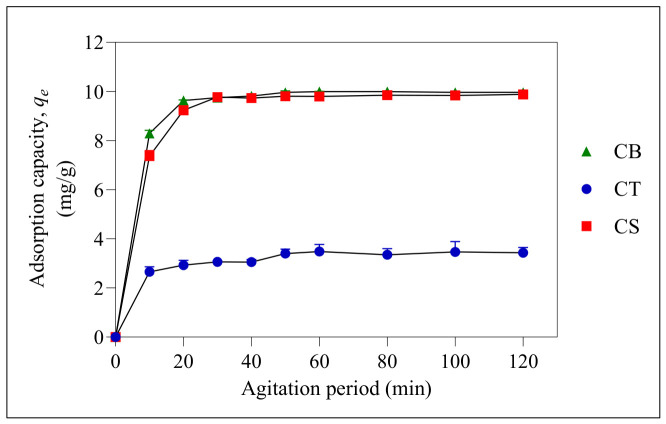
Effect of agitation period on the adsorption process at initial pH 6. Error bars represent the standard deviation obtained for triplicate measurements.

**Figure 7:**
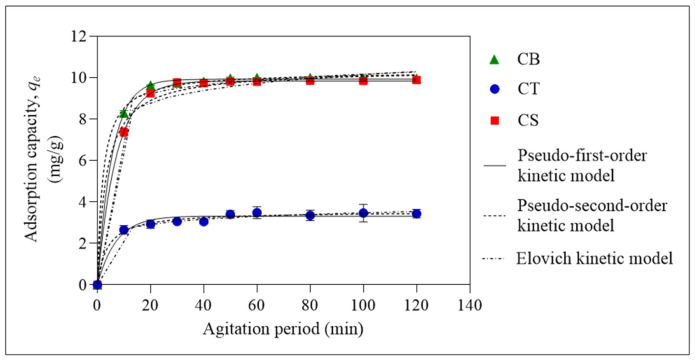
Pseudo-first-order (PFO), pseudo-second-order (PSO), and Elovich kinetic models plot for the adsorption of ER at initial pH 6. Error bars represent the standard deviation obtained for triplicate measurements.

**Figure 8:**
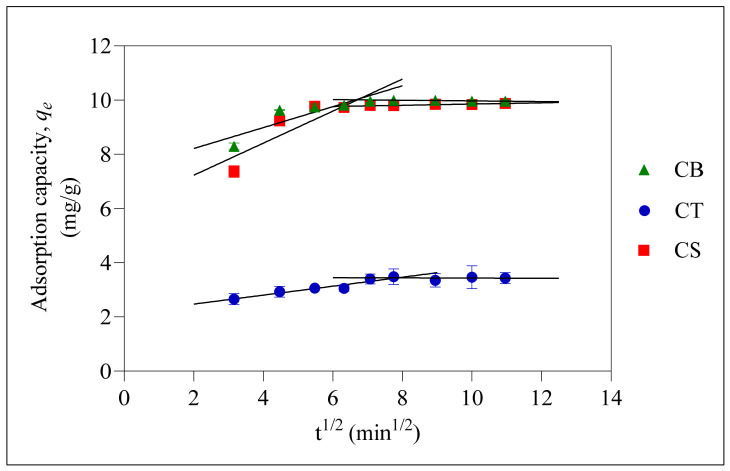
Intraparticle diffusion (IPD) model plot for the adsorption of ER onto CB, CT, and CS beads at initial pH 6. Error bars represent the standard deviation obtained for triplicate measurements.

Based on Figure 6, the adsorption capacity of all three beads increased with the agitation period. The optimum agitation period required for CS and CT beads to achieve equilibrium was 60 min, while CB beads required only 50 min. Based on the graph, the adsorption process can be divided into two stages. The initial stage is represented by the drastic increase in the adsorption capacity, as observed for CB, CT and CS beads. This is attributed to the availability of active sites for the adsorption of ER. This is followed by the second stage, where the adsorption capacity plateaus due to the exhaustion of these active sites. The non-linear pseudo-first-order (PFO), pseudo-second-order (PSO), intraparticle diffusion (IPD), and Elovich kinetic models were used to analyse the kinetic data obtained for CB, CT, and CS beads, as depicted in Figure 7 and Figure 8 and Table 1. These models provide insight into the rate-controlling step governing adsorption and are important for designing efficient wastewater treatment systems [51]. Non-linear models were used because there are various linear forms for each kinetic model, and linearisation can introduce error functions that may affect the data accuracy [52,53].

The first model used to analyse the kinetic data was the PFO kinetic model. According to this model, the rate-controlling step during adsorption is predominantly governed by physical adsorption, with no interactions between the adsorbed molecules [54]. The CB, CT and CS beads showed a good fit with this model, with a correlation of determination (*R*^2^) of more than 0.95, indicating that physisorption was involved in the rate-controlling step.

All three beads also showed excellent agreement with the PSO kinetic model. This model suggests that the rate-controlling step involves chemisorption through the sharing or exchange of electrons, involving valence forces. It also assumes that adsorption occurs proportionately to the number of active sites available on the adsorbent surface [55]. Technically, this model is applicable throughout the contact time, in comparison to PFO, which is normally applicable at the beginning of the adsorption process.

The CB, CT, and CS beads showed good agreement with the Elovich model, with a coefficient of determination (*R*^2^) value higher than 0.95. The Elovich model indicates that the adsorbent surface is energetically heterogeneous and that the controlling rate step also involves chemical interactions [56]. Among the three beads, the rate constant, *α*, and activation energy, *β*, for CT beads were the highest, consistent with the PSO analysis.

The plot in Figure 8 is based on the linear Weber and Morris kinetic model, also known as the IPD model. The plots did not pass through 0, indicating that intraparticle diffusion was not the sole rate-controlling step during adsorption [57]. The plot showed two stages, indicating that two steps were involved during the adsorption process. The first slope represents the progressive adsorption involving intraparticle diffusion as the rate-controlling step, whereby the adsorbate migrates from the solution into the adsorbent pores. This is followed by a slower adsorption stage, whereby the adsorbate is transported into the inner part of the adsorbent [58]. The *k_int,_*_1_ constant represents the rate of intraparticle diffusion, and all three beads displayed higher *k_int,_*_1_ values, indicating that adsorption of ER was controlled by intraparticle diffusion [59]. The *c* constant obtained from the IPD model in the second stage was higher than in the first stage, suggesting a larger boundary layer thickness during the second stage, corresponding to the plateau in adsorption capacity [60]. Overall, the rate-controlling step for the adsorption of ER onto CB, CT and CS beads involves the initial intraparticle diffusion step followed by chemisorption and physisorption.

#### 2.2.3. Effect of Adsorbent Dosage

**Figure 9:**
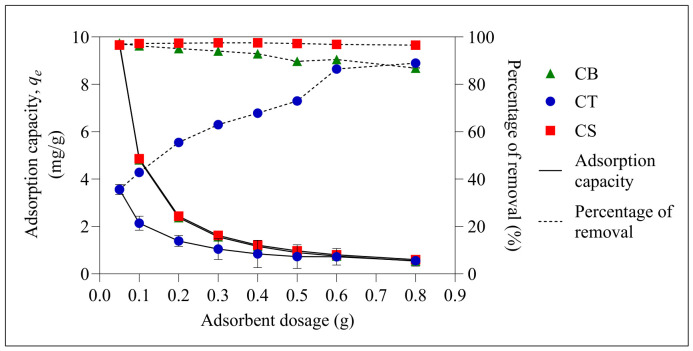
Effect of adsorbent dosage on the adsorption capacity and percentage removal of ER at initial pH 6. Error bars represent the standard deviation obtained for triplicate measurements.

The adsorption capacity for all three beads decreased as the dosage was increased (Figure 9). This is because, as the dosage increased, the number of available sites also increased. However, since the number of adsorbates in the solution remained constant, many adsorption sites remained unoccupied during the adsorption process, thereby reducing the adsorption capacity of the beads [61]. On the other hand, the percentage removal for CB and CS beads reached the optimum level at a lower dosage, while CT beads showed a gradual increase in percentage removal as the dosage increased. The CB and CS beads exhibited a slight reduction in percentage removal at higher adsorbent dosages due to potential particle aggregation, which reduces the surface area of the adsorbent. A much higher adsorbent dosage was not used in this study, as it may cause intermolecular crowding, thereby limiting the binding ability of the adsorbent [62]. The optimum adsorbent dosages selected for CB, CT, and CS beads were 0.05 g, 0.60 g, and 0.20 g, respectively.

### 2.3. Adsorption Isotherm

**Figure 10:**
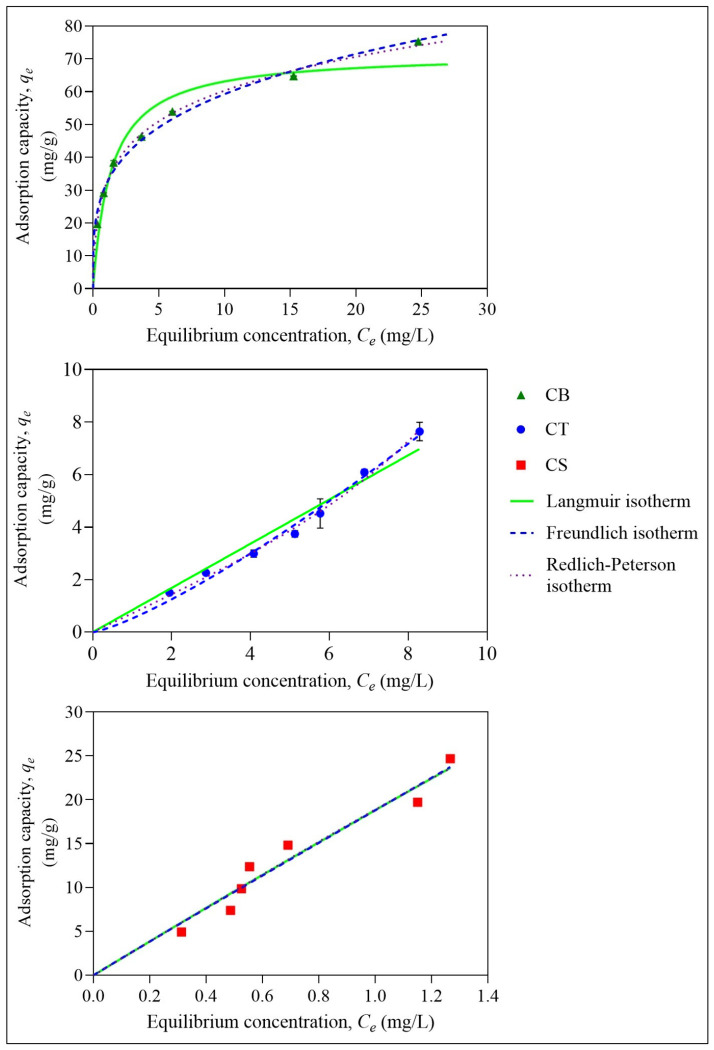
Langmuir, Freundlich, and Redlich–Peterson isotherms for the adsorption of ER onto CB, CT, and CS beads at initial pH 6. Error bars represent the standard deviation obtained for triplicate measurements.

The isotherm models are crucial in providing information on the interaction between the adsorbate and the adsorbent. As the interaction could be physical or chemical, three isotherm models were used to characterise the adsorption data, as shown in Figure 10 and Table 2.

The Langmuir isotherm model is the most prominent model used in the study of adsorption, whereby the maximum adsorption capacity of adsorbents obtained through this model can be used to compare with the efficiency of other adsorbents. It represents the chemisorption at the monolayer and homogeneous adsorption sites. According to this model, once an adsorbate has occupied an adsorption site, no further adsorption takes place at the same site [63]. Some of the possible interactions that can take place are ion-exchange, surface complexation, or precipitation mechanisms [62]. Based on Table 2, the *R*^2^ obtained for all three beads was lower than 0.95; therefore, it shows a moderate fit with this model. The highest capacity, *V_m_*, was recorded by the CS beads with 120.30 mg/g, followed by the CT beads with 89.33 mg/g and finally by the CB beads with 71.80 mg/g. The Langmuir constant related to the affinity of the binding sites, *b_L_*, showed that CB beads had the highest affinity for ER, although the maximum capacity obtained was the lowest. The low adsorption capacity is aligned with the surface area obtained for this bead [64]. To further evaluate the favourability of the adsorption process, the dimensionless constant factor, *R_L_*, was calculated, and it was found that the values were between 0 and 1, showing that the adsorption process was favoured throughout the concentration studied for all three beads [65].

Meanwhile, based on the Freundlich isotherm model, the CB and CT beads showed the highest agreement with it. The lowest agreement was shown by the CS beads. Based on this model, the adsorbent consists of a heterogeneous surface and undergoes a multilayer physisorption process [66]. Based on the *n* value, which represents the intensity of adsorption, CB beads have the highest value, implying that CB beads have a high intensity for the adsorbate, agreeing with the Langmuir model [65]. Furthermore, in comparison with the Langmuir isotherm, the Freundlich isotherm showed a better fit for all three beads, implying a heterogeneous surface with a multilayer adsorption process. Some of the possible interactions that can contribute to multilayer physisorption are electrostatic interactions, hydrogen bonding and van der Waals forces.

The next isotherm model used in this study was the Redlich–Peterson isotherm model. This model can be applied regardless of the nature of the adsorbent, whether it is homogeneous or heterogeneous. This model is versatile, as it incorporates both the Langmuir and the Freundlich models. This model can convert to the Freundlich isotherm at high adsorbate concentrations, while at lower concentrations of adsorbate, it agrees with the Langmuir isotherm [67]. The highest agreement with this isotherm is provided by CB beads. Both CT and CS beads have an *R*^2^ of 0.947. Based on this model, it was found that the *g* value for CB and CT beads was less than one, indicating the process is leaning towards the Freundlich isotherm, agreeing with the finding from the Freundlich model. However, for CS beads, the *g* value is more than 1, indicating the data do not agree with either the Langmuir or Freundlich isotherm.

The isotherm models suggest that the adsorption of ER onto CB and CT beads involves multilayer physisorption through relatively weak interactions, such as electrostatic interaction between the positively charged adsorbent and the negatively charged ER, and hydrogen bonding between the amine groups of chitosan and the oxygen-containing groups on ER. In contrast, the adsorption process for CS beads appears more complex and requires further investigation.

A comparison of the theoretical maximum adsorption capacities, based on the Langmuir isotherm, for various adsorbents is presented in Table 3. The performance of CB, CT, and CS is comparable to that of other adsorbents. Furthermore, the preparation technique used for CB, CT and CS was relatively straightforward, yet these materials were able to achieve performance comparable to adsorbents derived from metal–organic frameworks, nanoparticles, activated carbon, multi-walled carbon nanotubes, and graphene oxide decorated with metal oxides.

### 2.4. Mechanism of Adsorption

The adsorption of dye onto the adsorbent begins with diffusion from the bulk liquid to the adsorbent surface. Subsequently, the dye molecules interact with the adsorbent via physical interactions such as electrostatic interaction, hydrogen bonding, and π-π interactions or through chemical interactions involving electron sharing and formation of valence bonds. The binding process can be reversible if the interaction is weak or irreversible if the interaction is strong [70].

At an acidic pH, the amine groups in chitosan are protonated, forming the -NH_3_^+^. The STTP, once dissolved in water, forms tripolyphosphate (TPP) (P_3_O_10_^5−^) ions, which can undergo hydrolysis to form different protonated species (H_4_P_3_O_10_^−^, H_3_P_3_O_10_^2−^, H_2_P_3_O_10_^3−^, and HP_3_O_10_^4−^). Each form has its own pKa value, with P_3_O_10_^5−^ having the pKa value of 9.24 [71]. The -NH_3_^+^ groups on chitosan and P_3_O_10_^5−^ ions from STPP will form ionic crosslinking; however, the effect of the ionotropic gelation process depends on the distribution of species present in the solution. As the pH of the STPP solution was not controlled in this study, it is assumed that P_3_O_10_^5−^ was the predominant ion present. According to Kourkaras et al. [72], the P_3_O_10_^5−^ can accept protons from chitosan, thereby interacting with the -NH_3_^+^ groups of chitosan.

On the other hand, depending on the pH of the solution, SM undergoes hydrolysis once dissolved in water to form sulphur dioxide (SO_2_), bisulphite (HSO_3_^−^), or sulphite (SO_3_^2−^) [73]. As the pH was not controlled during the preparation of the beads, sulphite ions are expected to be more prevalent in the solution. The possible interaction during the crosslinking process involves the -NH_3_^+^ on chitosan and the SO_3_^2−^ ions from SM. Meanwhile, ER at pH 5 and 6 exists in monoanionic and dianionic structures due to the formation of COO^−^ and O^−^ groups.

The molecular electrostatic potential (MEP) was used to visualise and analyse the distribution of electrostatic charge on ER, chitosan, tripolyphosphate, sulphite and bisulphite. It provides insights into the reactivity and interaction sites of molecules by representing the electrostatic potential on molecular surfaces. MEP maps help in understanding the regions where molecules are likely to undergo chemical reactions or attract other molecules based on charge distribution [74].

**Figure 11:**
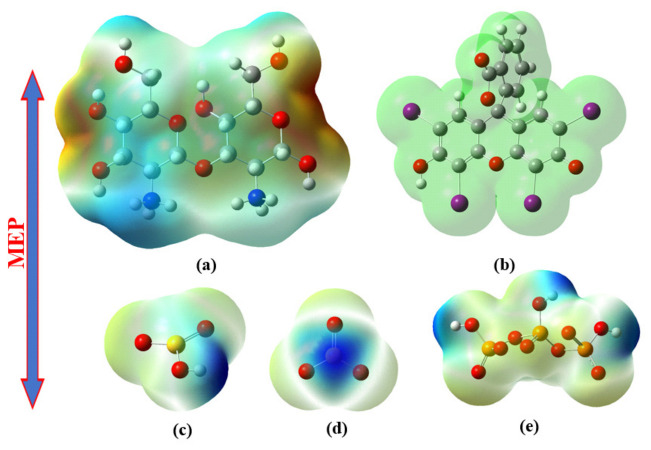
Distribution of the electron density on the optimal molecular geometries of (**a**) chitosan, (**b**) ER, (**c**) bisulphite, (**d**) sulphite, and (**e**) tripolyphosphate ions. (Colour coding for atoms: white-hydrogen; grey-carbon; red-oxygen; blue-nitrogen; purple-iodine; yellow-sulphur; orange-phosphorus).

Figure 11 depicts the MEPs of CB, ER, sulphite (SO_3_^2−^), bisulphite (HSO_3_^−^) and tripolyphosphate (P_3_O_10_^5−^) ions. The MEP surface of ER is relatively uniform, although the oxygen atoms are associated with slightly more negative electrostatic potential than the surrounding regions, consistent with their electron-rich character. In CB beads, the protonated amine groups (-NH_3_^+^) correspond to regions of positive electrostatic potential [75]. This suggests that adsorption may be driven by electrostatic interactions between the oxygenated groups of ER (COO^−^ and O^−^), and the protonated amine groups of chitosan [76]. Furthermore, hydrogen bonding may also occur between the hydrogen donor from -NH_3_^+^ groups of chitosan and the oxygen atoms in COO^−^ or O^−^ groups of ER [77].

**Figure 12:**
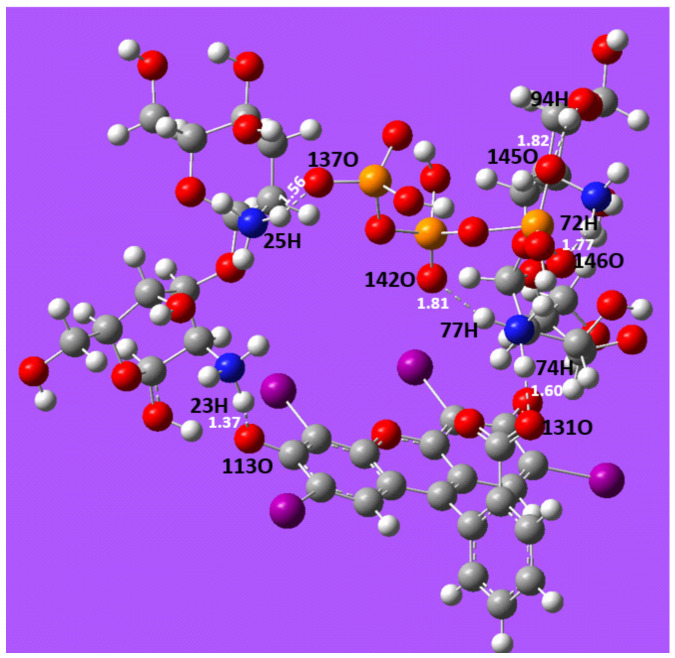
Molecular interaction between CT and ER following the adsorption process. (Colour coding for atoms: white-hydrogen; grey-carbon; red-oxygen; blue-nitrogen; purple-iodine; orange-phosphorus).

The distance between ER and CT and CS was determined using the optimised structures of the CT/ER and CS/ER complexes with the LanL2DZ/WB97XD basis sets. In Figure 12, the optimal molecular structure of the CT/ER complex is shown. Adsorption of ER occurs through the formation of a hydrogen bond (1.37 Å) between the oxygen ion (-O^−^) of ER and the hydrogen atom of the protonated amine groups (-NH_3_^+^) in CT beads. The oxygen ion (-COO^−^) in ER also interacts with the hydrogen atom of the protonated amine group in CT, forming a hydrogen bond at 1.60 Å. This suggests that the adsorption of ER onto CT beads is primarily driven by weak interactions between the oxygenated groups of ER and the nitrogen-containing groups of CT beads. 

**Figure 13:**
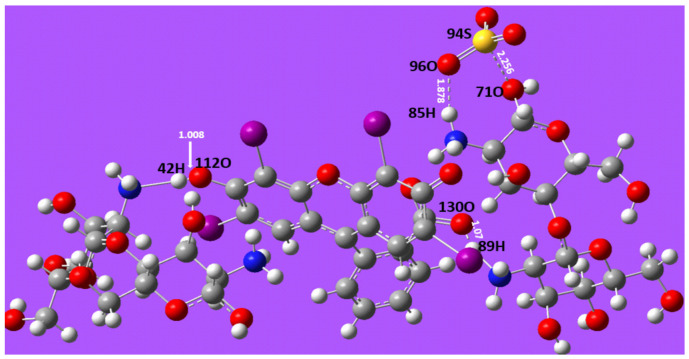
Molecular interaction between CS and ER following the adsorption process. (Colour coding for atoms: white-hydrogen; grey-carbon; red-oxygen; blue-nitrogen; purple-iodine; yellow-sulphur).

In Figure 13, the optimised molecular structure of the CS/ER complex is presented. Similar to the CT/ER complex, the adsorption of ER occurs through hydrogen bond formation (smallest distance: 1.01 Å) between the oxygen ion (-O^−^) in ER and the hydrogen atom in the protonated amine group (-NH_3_^+^) of CS beads. The bond distance between -COO^−^ in ER and -NH_3_^+^ in chitosan was 1.07 Å. This suggests that the adsorption mechanism on the CS surface is also driven by weak interactions between the oxygenated group in ER and the nitrogenous groups of CS beads [78]. It was found that the bond length involved in the CS/ER interaction was shorter than that in CT/ER interaction. Therefore, the formation of the CS/ER complex correlates with the higher adsorption capacity attained by CS for the adsorption of ER [79,80]. 

Formation energy or interaction energy plays a crucial role in predicting the stability of a complex in chemical systems [81]. The interaction energies were calculated based on the total energy. The formation energies for CS/ER and CT/ER were −3.11 a.u. and −3.46 a.u., respectively. A negative interaction energy implies that the complex formation is energetically favourable, with the system releasing energy during the process. A more negative energy value indicates a more stable complex [82,83]. The CS/ER showed a lower interaction energy, consistent with the highest adsorption capacity attained by CS beads.

**Figure 14:**
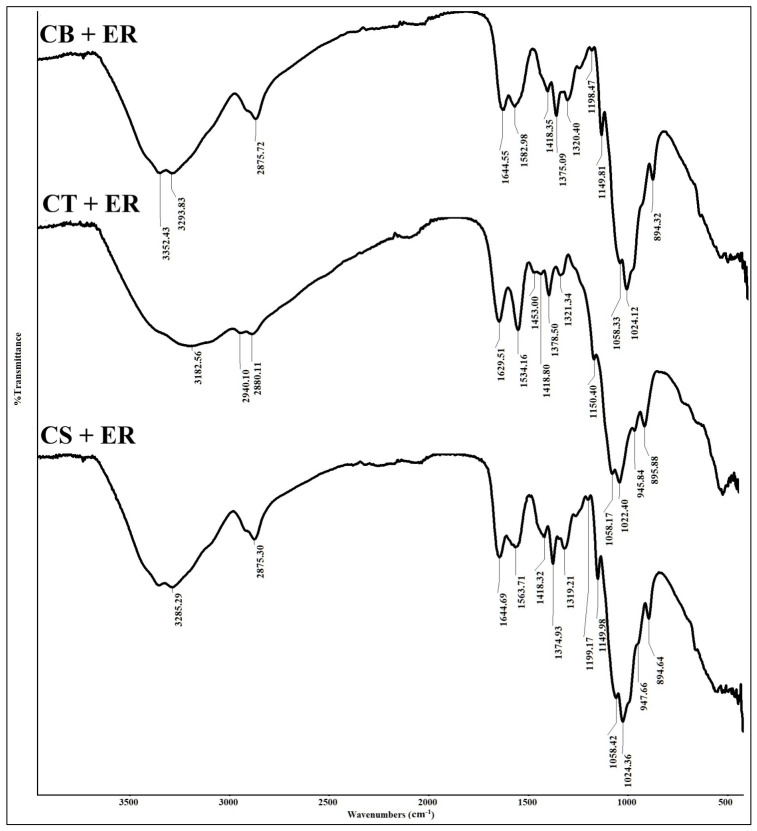
FTIR spectra of CB, CT, and CS beads after the adsorption of ER.

To further substantiate the adsorption mechanisms, FTIR analysis was carried out on all three beads after the adsorption, and the spectra are shown in Figure 14. After the adsorption of ER, all beads showed changes in FTIR bands. The broadband associated with -OH and -NH stretching at 3182 cm^−1^ became broader in all three beads, suggesting successful adsorption of ER. This can be attributed to the interaction between COO^−^ or O^−^ groups of ER and the amine functional groups of chitosan. In addition, CB and CS beads showed a marked reduction in the intensity of N-H bending vibrations (1582 cm^−1^ and 1563 cm^−1^), indicating the involvement of -NH groups in the adsorption process. Furthermore, the band assigned to C-N stretching at 1375 cm^−1^ exhibited increased intensity after adsorption on all three beads, further confirming the involvement of amine groups in the chitosan structure. This observation aligns with DFT findings. The peak at 1646 cm^−1^, representing C=O, became more intense after adsorption of ER in all three beads, suggesting its involvement during the adsorption process. In the CT and CS beads, the intensity of the bands at 1378 cm^−1^ and 1557 cm^−1^, respectively, increased, indicating the presence of protonated amine groups during adsorption.

**Figure 15:**
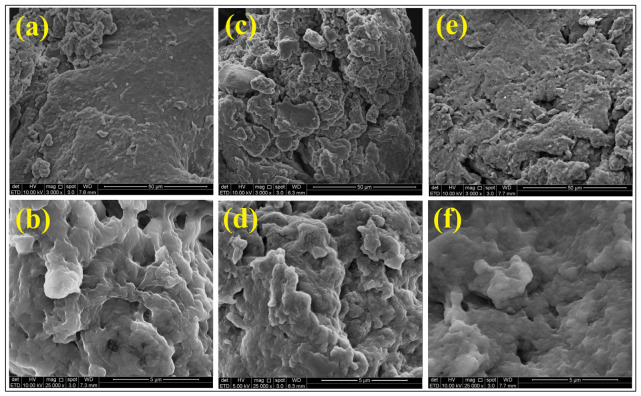
SEM of (**a**,**b**) CB, (**c**,**d**) CT, and (**e**,**f**) CS beads at 3000× magnification and 25,000× magnification after the adsorption of ER.

**Figure 16:**
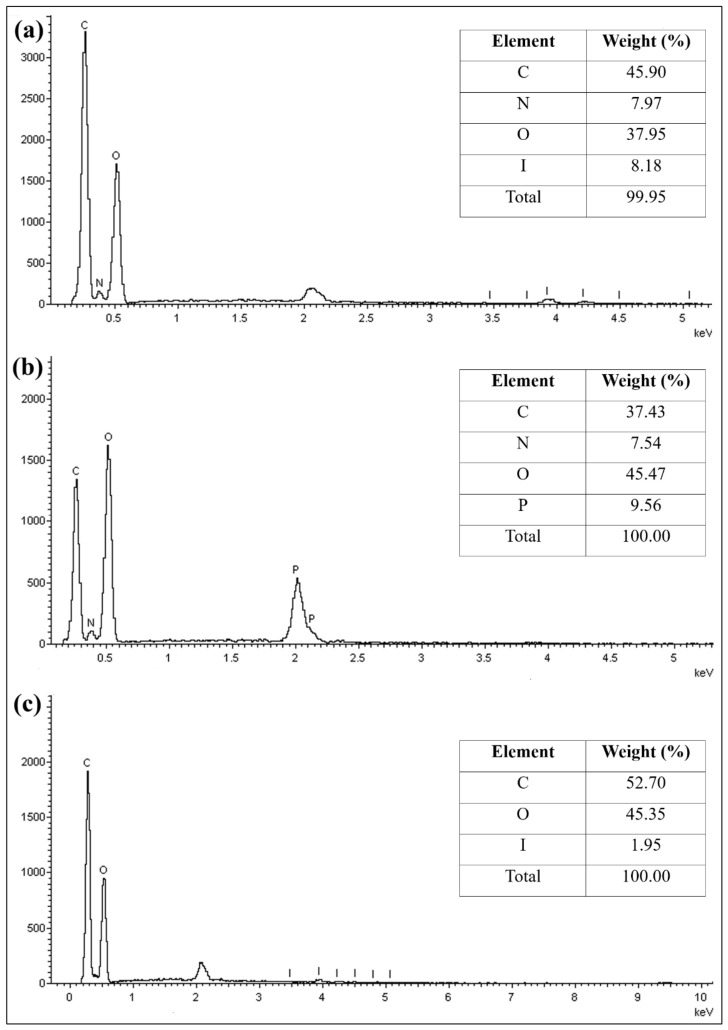
EDS spectra of (**a**) CB, (**b**) CT, and (**c**) CS beads after the adsorption of ER.

The SEM images of CB, CT and CS beads after the adsorption of ER (Figure 15) showed noticeable differences. The surfaces appeared highly uneven and rough, with visible pores on the surface of the beads. This indicates morphological changes after the adsorption of ER. The EDS analysis further confirmed the presence of ER on the CB and CS through the detection of iodine (I), as shown in Figure 16. The CT beads did not show the presence of iodine, likely due to a lower adsorption capacity. Overall, the analysis confirms the involvement of protonated amine groups during the adsorption of ER. Based on the DFT analysis, it is proposed that the hydrogen bonding between oxygenated groups in ER and nitrogenous groups in chitosan governs the adsorption process.

## 3. Materials and Methods

### 3.1. Materials

Chitosan, procured from Sigma-Aldrich, Malaysia (country of origin: USA), has a degree of deacetylation of 60.9%. ER was purchased from Sigma-Aldrich, Malaysia (country of origin: India). The chemicals used for adsorbent preparation, namely STPP, SM, and sodium hydroxide (NaOH), were obtained from R&M Chemicals, Selangor, Malaysia. Glacial acetic acid (99.8%, CH_3_COOH) and hydrochloric acid (HCl) (37.0%) were purchased from Systerm, Selangor, Malaysia. All reagents utilised throughout the study were of analytical grade. All preparations were carried out at room temperature.

### 3.2. Preparation of Chitosan (CB) Beads

A 3.33% (*w*/*v*) chitosan solution was produced by dissolving chitosan powder in 5% (*v*/*v*) acetic acid. The prepared solution was left to stand for 24 h before being introduced dropwise into a 0.5 M NaOH solution under continuous stirring at 200 rotations per minute (rpm). Formation of white beads indicated successful gelation. The resulting beads were filtered using a strainer, washed thoroughly with distilled water, and left to air dry. Once dried, the beads were ground and sieved to obtain a uniform particle size of 250 μm. These prepared beads are hereafter referred to as CB beads.

### 3.3. Preparation of Chitosan–Tripolyphosphate (CT) Beads

CT beads were synthesised using a modified approach adapted from the method reported by Fanaee and Filiaggi [84]. A 3.33% (*w*/*v*) chitosan solution was first prepared in 5% (*v*/*v*) acetic acid and allowed to stand overnight to ensure complete dissolution. The resulting viscous solution was introduced dropwise into a 0.05 M STPP solution under continuous stirring at 200 rpm. The gelled spheres formed were subsequently collected by filtration using a strainer, rinsed with distilled water, and air-dried. The dried beads were ground to obtain particles smaller than 250 μm. Hereafter, the beads are referred to as CT beads.

### 3.4. Preparation of Chitosan–Sulphite (CS) Beads

For CS bead preparation, the same 3.33% (*w*/*v*) chitosan solution in 5% (*v*/*v*) acetic acid was prepared and left overnight. The solution was then blended with 0.05 M SM to form a homogeneous mixture. The mixture was then added dropwise into 0.5 M NaOH solution under continuous stirring at 200 rpm, which resulted in the formation of spherical, white and elastic beads. The beads were then filtered using a strainer, rinsed with distilled water and air-dried. Finally, the formed CS beads were ground to achieve a particle size less than 250 μm. These beads are referred to as CS beads.

### 3.5. Analytical Instrumental Analyses of CB, CT and CS Beads

The chemical structure of all three adsorbents was analysed using a Nicolet Summit FTIR Spectrometer (ThermoFisher Scientific, MA, USA) with the potassium bromide (KBr) technique. The spectra were recorded from 4000 to 400 cm^−1^, with 32 scans at a resolution of 4 cm^−1^. The surface morphology was examined using a field emission scanning electron microscope (FESEM) (ZEISS Merlin, Oberkochen, Germany) coupled with energy-dispersive spectroscopy (EDS) (Oxford Xmax50, Oxford Instruments, High Wycombe, UK). The images were captured at an operating voltage of 5–10 kV. Textural properties, including the surface area and porosity, were determined using a ASAP 2020 Accelerated Surface Area and Porosimetry system (Micromeritics, GA, USA) using nitrogen (N_2_) gas. The concentration of ER dye before and after adsorption was measured using a double-beam UV–Visible spectrophotometer (UV-1900i Plus, Shimadzu, Kyoto, Japan).

### 3.6. Batch Adsorption Experiments

The batch adsorption experiments were carried out using different concentrations of ER. The effect of pH on the molecular structure of ER was observed based on the full scan spectrum using a UV–Vis spectrophotometer. The solutions were adjusted to the appropriate pH either by using diluted NaOH or diluted HCl. Three critical parameters were optimised, namely the initial pH, contact time, and adsorbent dosage. These three parameters played crucial roles in influencing the adsorption capacity of the adsorbents; therefore, they were selected for this study. The optimisation experiments were conducted using 50 mL of 10 mg/L ER for 30 min. The adsorbent dosage used is 0.05 g, unless stated otherwise. The agitation rate used throughout the study was 150 rpm. After the respective contact time, the solution was filtered using Whatman filter paper no.1 and scanned at 527 nm using the UV–Visible spectrophotometer. In order to attain precision, all adsorption experiments were conducted in triplicate, and the graphs show error bars representing the standard deviation. The experimental errors in this study were below ±2%. The adsorption capacity, *q_e_* (mg/g), of the adsorbents was calculated based on this equation:

(1) $$q_{e}=\frac{C_{o}-C_{e}}{m}V\;$$

where *C_o_*, *C_e_*, *V* and *m* represent the initial concentration before the adsorption process (mg/L), the concentration after the adsorption process (mg/L), the volume of ER used (L) and the weight of the beads used (g), respectively. The initial pH study was conducted from pH 5.0 to 10.0, while the agitation period ranged from 10 to 120 min. The adsorbent dosage study was conducted in the range of 1.0 g/L to 16.0 g/L. Once all three parameters were finalised, the isotherm study was carried out using ER dye concentrations from 10 mg/L to 100 mg/L of ER dye.

The data from the agitation period study were analysed using three kinetic models. The non-linear PFO, PSO and Elovich models used are as follows [63,85]:

(2) $$q_{t}=q_{e}\left(1-{exp}^{-k_{1}t}\right)\;$$

(3) $$q_{t}=\frac{k_{2}q_{e}^{2}t}{1+k_{2}q_{e}t}$$

(4) $$q_{t}=\frac{1}{\beta }ln(1+\alpha \beta t)$$

where *q_t_*, and *q_e_* represent the adsorbent capacity (mg/g) at time *t* (min) and at equilibrium. The *k*_1_ (min^−1^), *k*_2_ (g/mg min), α (mg/g min) and β (g/mg) represent the PFO and PSO rate constants, the rate constant for chemical adsorption and the activation energy for chemical adsorption, respectively.

The isotherm data were analysed using Langmuir, Freundlich and Redlich–Peterson models. These models are represented as follows [57,85].

(5) $$q_{e}=\frac{b_{L}V_{m}C_{e}}{1+b_{L}C_{e}}$$

(6) $$q_{e}=K_{F}C_{e}^{1/n}$$

(7) $$q_{e}=\frac{AC_{e}}{1+BC_{e}^{g}}$$

where *C_e_* represents the concentration of ER at equilibrium (mg/L), *q_e_* represents the adsorption capacity (mg/g), *b_L_* is the equilibrium constant related to the affinity of the binding sites (L/mg), *V_m_* is the maximum adsorption capacity of the adsorbent at the monolayer (mg/g), *K_F_* is the constant representing the adsorption capacity ((mg/g)(L/m)^1/*n*^), and *n* is an empirical parameter related to the intensity of adsorption, which changes according to the heterogeneity of the adsorbent. *A* and *B* represent the Redlich–Peterson constant (L/g and (L/mg)^1/*n*^), and *g* represents the exponent related to the heterogeneity factor, which lies between 0 and 1. All kinetic and isotherm models were analysed using Prism 10 (Version 10.6.0) and MATLAB R2020a (Curve Fitting Toolbox) (9.8.0.1323502). The accuracy of the fit was estimated based on the correlation of determination (*R*^2^) and root mean square errors (RMSE). An *R*^2^ of more than 0.95 is considered a good fit with the model. The DFT method was used to predict the possible adsorption mechanism that took place during the adsorption of ER. It explores the interaction energy, structural and electronic aspects of the beads and ER. Gaussian 09W 9.5 (Revision D.01) and GaussView 6.0.16 were used to optimise the molecular structure of the adsorbate and adsorbents and to perform the calculation using the LanL2DZ/WB97XD method.

## 4. Conclusions

The CB, CT and CS beads were successfully prepared, and the FTIR spectra confirmed that substantial crosslinking had taken place. BET surface analysis showed that CS beads had the highest surface area (0.84 m^2^/g) with an average pore diameter of 41.21 Å, while CT beads recorded the lowest surface area and pore diameter. The adsorption process was optimised based on three critical parameters: the initial pH of ER, the agitation period and the adsorbent dosage. The data obtained from the agitation period were analysed using three well-established kinetic models. Based on the analysis, it was found that the rate of the adsorption process for all three beads was not solely dependent on film intraparticle diffusion. Instead, chemisorption and physisorption played important roles, along with diffusion of the adsorbate into the pores, as suggested by the Elovich model as well. Based on the Langmuir isotherm, the CS attained the highest adsorption performance, indicating the potential of SM as a favourable crosslinking agent compared to STPP. This finding was consistent with the higher surface area and pore diameter recorded for CS beads. The overall conclusion from the isotherm models showed that CB and CT leaned towards the Freundlich isotherm, implying multilayer adsorption on a heterogeneous surface, although chemisorption remained relevant based on the kinetic findings. The adsorption process for CS beads suggested a more complex system requiring further investigation. DFT analysis indicated that adsorption involved weak interactions between the ionised -O- and -COO^−^ groups of ER and the protonated amine groups of the beads. Overall, this study successfully compared the performance of CB, CT and CS beads and highlighted the potential of SM as an alternative crosslinking agent to enhance chitosan biosorbent performance. This work aligns with the United Nations Sustainable Development Goal 6 by contributing to improved water quality. Future research can be extended to include thermodynamic studies and real wastewater systems, as well as exploring higher crosslinking ratios and different kinetic and isotherm models to better understand the adsorption mechanism. Furthermore, other analytical techniques such as X-Ray Photoelectron Spectroscopy (XPS) can be employed to provide deeper insight into the interactions occurring during the adsorption process.

## Figures and Tables

**Table 1:** Kinetic parameters for the adsorption of ER onto chitosan (CB), chitosan–tripolyphosphate (CT), and chitosan–sulphite (CS) beads at initial pH 6.

Adsorbents	CB	CT	CS
***q_e,exp_*** **(mg/g)**	9.97	3.49	9.80
**Pseudo-first-order**			
*q_e,cal_* (mg/g)	9.93	3.32	9.84
*k*_1_ (min^−1^)	1.79 × 10^−1^	1.41 × 10^−1^	1.39 × 10^−1^
*R* ^2^	1.000	0.976	1.000
RMSE	7.221 × 10^−2^	1.73 × 10^−1^	4.59 × 10^−2^
**Pseudo-second-order**			
*q_e,cal_* (mg/g)	10.29	3.55	10.39
*k*_2_ (g/mg min)	4.70 × 10^−2^	7.43 × 10^−2^	2.86 × 10^−2^
*R* ^2^	0.998	0.991	0.993
RMSE	1.64 × 10^−1^	9.99 × 10^−2^	2.59 × 10^−1^
**Intraparticle diffusion**			
*k_int_*_,1_ (mg/g min^½^)	3.86 × 10^−1^	1.66 × 10^−1^	5.92 × 10^−1^
*c* (mg/g)	7.44	2.14	6.05
*R* ^2^	0.761	0.898	0.766
*k_int_*_,2_ (mg/g min^½^)	−1.25 × 10^−2^	−3.80 × 10^−3^	2.24 × 10^−2^
*c* (mg/g)	10.10	3.47	9.63
*R* ^2^	0.781	0.008	0.816
**Elovich**			
*α* (mg/g min)	83.99	91.68	87.86
*β* (g/mg)	8.33 × 10^−1^	2.93	8.59 × 10^−1^
*R* ^2^	0.967	0.991	0.973
RMSE	5.96 × 10^−1^	1.04 × 10^−1^	5.43 × 10^−1^

**Table 2:** Langmuir, Freundlich, and Redlich–Peterson isotherm parameters for the adsorption of ER onto CB, CT, and CS beads at initial pH 6.

Parameter	CB	CT	CS
**Langmuir**			
*V_m_* (mg/g)	71.80	89.33	120.3
*b_L_* (L/mg)	7.28 × 10^−1^	9.99 × 10^−3^	1.84 × 10^−1^
*R* ^2^	0.933	0.943	0.938
RMSE	4.80	0.57	1.90
**Freundlich**			
*K_F_* (mg/g)	31.78	5.22 × 10^−1^	18.81
*n*	3.70	7.93 × 10^−1^	1.02
*R* ^2^	0.987	0.986	0.947
RMSE	2.16	0.28	1.77
**Redlich**–**Peterson**			
*A* (L/g)	180.20	22.11	19.27
*B* (L/mg)^1/*n*^	4.66	23.20	2.05 × 10^−2^
***g***	7.93 × 10^−1^	3.88 × 10^−2^	2.32
*R* ^2^	0.996	0.947	0.947
RMSE	1.53	0.61	1.96

**Table 3:** Comparison of adsorption capacities of various adsorbents for ER removal.

Adsorbent	Adsorption Capacity (mg/g)	Experimental Conditions		Ref.
		Optimum pH	Concentration Studied (mg/L)	
Fe-Co-V supported on zeolite nanostructure	1.82	9	10–40 mg/L	[16]
PVDF modified with MnFe-layered double hydroxides	6.14	5	7–19 mg/L	[68]
Corn cob	7.50	6	13 to 309 mg/L	[14]
Carbonised dates	9.09	7	20–300 mg/L	[15]
Wood chip	25.2	6	13 to 309 mg/L	[14]
Chitosan without ER imprinting	39.06	6	Not provided	[26]
CB beads	71.80	6	10–100 mg/L	This study
CT beads	89.33	6	10–100 mg/L	This study
ER-imprinted magnetic chitosan	116.27	6	Not provided	[26]
CS beads	120.30	6	10–100 mg/L	This study
Activated carbon loaded with zinc oxide	144.92	3	50–200 mg/L	[12]
Decorated graphene oxide with magnetic iron oxide nanoparticles	149.25	7	30, 35 and 40 µg/mL	[69]
Multiwalled carbon nanotubes decorated with zinc oxide and silver oxide	184.94	3	100–250 mg/L	[13]
Chitosan–alginate biocomposite crosslinked with ECH and STPP	319.00	6.5	10–1000 mg/L	[23]

## Data Availability

The original contributions presented in this study are included in the article. Further inquiries can be directed to the corresponding author.
